# Nitrogen Use and Carbon Sequestered by Corn Rotations in the Northern Corn Belt, U.S.

**DOI:** 10.1100/tsw.2001.90

**Published:** 2001-09-28

**Authors:** Joseph L. Pikul, Thomas E. Schumacher, Merle Vigil

**Affiliations:** USDA-ARS, Northern Grain Insects Research Laboratory, Brookings, SD 57006, USA

**Keywords:** soybean, wheat, alfalfa, crop rotation, soil quality, nitrogen value, nitrate nitrogen, soil carbon, Brookings, South Dakota

## Abstract

Diversified crop rotation may improve production efficiency, reduce fertilizer nitrogen (N) requirements for corn (Zea mays L.), and increase soil carbon (C) storage. Objectives were to determine effect of rotation and fertilizer N on soil C sequestration and N use. An experiment was started in 1990 on a Barnes clay loam (U.S. soil taxonomy: fine-loamy, mixed, superactive, frigid Calcic Hapludoll) near Brookings, SD. Tillage systems for corn–soybean (Glycine max [L.] Merr.) rotations were conventional tillage (CS) and ridge tillage (CSr). Rotations under conventional tillage were continuous corn (CC), and a 4-year rotation of corn–soybean–wheat (Triticum aestivum L.) companion-seeded with alfalfa (Medicago sativa L.)–alfalfa hay (CSWA). Additional treatments included plots of perennial warm season, cool season, and mixtures of warm and cool season grasses. N treatments for corn were corn fertilized for a grain yield of 8.5 Mg ha (highN), of 5.3 Mg ha (midN), and with no N fertilizer (noN). Total (1990–2000) corn grain yield was not different among rotations at 80.8 Mg ha under highN. Corn yield differences among rotations increased with decreased fertilizer N. Total (1990–2000) corn yields with noN fertilizer were 69 Mg ha under CSWA, 53 Mg ha under CS, and 35 Mg ha under CC. Total N attributed to rotations (noN treatments) was 0.68 Mg ha under CSWA, 0.61 Mg ha under CS, and 0.28 Mg ha under CC. Plant carbon return depended on rotation and N. In the past 10 years, total C returned from above- ground biomass was 29.8 Mg ha under CC with highN, and 12.8 Mg ha under CSWA with noN. Soil C in the top 15 cm significantly increased (0.7 g kg) with perennial grass cover, remained unchanged under CSr, and decreased (1.7 g kg) under CC, CS, and CSWA. C to N ratio significantly narrowed (–0.75) with CSWA and widened (0.72) under grass. Diversified rotations have potential to increase N use efficiency and reduce fertilizer N input for corn. However, within a corn production system using conventional tillage and producing (averaged across rotation and N treatment) about 6.2-Mg ha corn grain per year, we found no gain in soil C after 10 years regardless of rotation.

### INTRODUCTION

Crop rotation, residue management, fertility management, and tillage management are key tools having a direct impact on sustainability of the soil resource, but a definitive statement cannot always be made about the effect of management on soil condition. Interactions of crop and soil management are clouded by variation within and between major land resource areas. Rarely can results from one climatic (or production) region be extrapolated to another. The length of time required for soil–crop rotation systems to reach a quasi-equilibrate state slows the advance of science and, consequently, slows acceptance and adoption of alternative production systems by producers. Understanding soil condition and trend is fundamental to understanding the interaction of cropping system and soil productivity, and to ultimately defining sustainability.

Sustainable agriculture has been defined as one that, over the long term, enhances environmental quality and the resource base on which agriculture depends, provides for basic human food and fiber needs, is economically viable, and enhances the quality of life for farmers and society as a whole[1]. Profit margins for production of most crops are very narrow, and producers seek sustainable cropping systems that provide consistent return on investment[2]. Generally, increased diversity of crops grown in rotation enhances sustainability of agriculture systems because crops grown in rotation, with similar off-farm inputs, have greater yield than those grown in monoculture[3,4,5]. Crop rotations that include legumes also increase soil N levels[6,7]. N has been considered one of the best crop-input investments that a farmer can make in terms of return on dollars spent. Bundy et al.[8] estimated that in the 12 states of the north central U.S., at least 4 million tons of N fertilizer are applied annually to corn at a cost of about $800 million. Thus, there is substantial justification to improve N management in the north central region of the U.S. Objectives of our research were to determine the effect of rotation and fertilizer N on soil C sequestration and N use in the northern Corn Belt.

### EXPERIMENTAL METHODS/PROCEDURES

#### Experimental Site

Our study was located on the Eastern South Dakota Soil and Water Research Farm near Brookings, SD, U.S. (44°19′ north latitude, 96°46′ west longitude, and 500-m elevation), on a Barnes soil with nearly level topography. Brookings is located in a transition zone between cool (frigid temperature regime) and warm (mesic temperature regime) prairies. Annual precipitation is 580 mm. Soils of this area are Udic Borolls to the north, Udic Ustolls to the south, and Typic Ustolls to the west. Thus, cool soil temperatures and limited soil water can affect adoption of alternative crop production or tillage methods. Prior to the start of the study (1990), the land was cropped to oat and barley (1972–1978), corn rotated with soybean (1979–1987), soybean (1988), and spring wheat (1989).

#### Experimental Design and Management

Whole plots (rotations) in the split-plot experiment were arranged as a randomized complete block with three replications. Split plots (subplots) were N management. All phases of each rotation were present every year. Crop rotations were (1) continuous corn (CC), (2) a 2-year rotation of corn and soybean (CS), and (3) a 4-year rotation of corn, soybean, spring wheat, and alfalfa (CSWA). In the 4-year rotation, spring wheat was used as a grain crop and as a companion crop to establish alfalfa. Plots were 30 m long by 30 m wide. Prior to 1996, primary tillage was with a moldboard or chisel plow in the fall of the year (conventional tillage). Primary tillage since 1996 was with a chisel plow. Tillage has been about 20 cm deep. In 1995 and 1996, wet weather conditions precluded fall tillage.

An additional tillage treatment using ridge tillage was used for corn and soybean (CSr). With the exception of primary tillage, the CSr plots were treated as the CS plots. The CSr plots received only row cultivation for both corn and soybean crops. Cultivation has maintained a raised seedbed on CSr plots. Rows were oriented in the east–west direction for both conventional tillage rotations and ridge tillage.

Seedbeds for corn and soybean under conventional tillage were prepared in spring using a tandem disk and field cultivator. Corn and soybean were no-till planted on the previous crop row under ridge tillage. Seeding date, rate, and variety were the same for all tillage and N treatments in a given year[9]. Depending on weather, seeding was as early as May 5 for corn and May 11 for soybean. Row spacing for corn and soybean was 76 cm. Both conventional and ridge tillage plots were cultivated twice during the early growing season for weed control in corn and soybean. Urea-N was side-dressed immediately before the second cultivation of corn. Pikul et al.[9] provide additional details of experimental design.

N treatments (subplots) termed high N, medium N, and low N were: corn fertilized for a yield goal (YG) of 8.5 Mg of grain per hectare (highN), corn fertilized for a YG of 5.3 Mg of grain per hectare (midN), and corn not fertilized (noN). A total soil nitrate (TSN) test was used to estimate fertilizer N prescription (NP) for corn[10]. On each N treatment, NP was calculated as

$$NP = 0.022YG - TSN \qquad (1)$$

Adjustment[10] to NP for previous crop or sampling date was not made. N prescription for each tillage and N treatment, expressed as an average of three replications, was met by applying starter fertilizer with the seed and side-dressing with appropriate amounts of urea as 46-0-0 (elemental N-P-K).

Starter fertilizer for both corn and soybean was applied at seeding and placed 5 cm to the side and 5 cm deeper than seed. Starting with the 1996 crop year, 112 kg ha$^{-1}$ of starter fertilizer as 14-16-11, 7-16-11, and 0-16-11 (elemental N-P-K) were applied on highN, midN, and noN plots, respectively. Prior to 1996, 111 kg ha$^{-1}$ of starter fertilizer were applied to highN, and 53 kg ha$^{-1}$ were applied to midN as 13-14-11 (elemental N-P-K). Starter fertilizer was not used on noN plots prior to 1996. Soil P levels were elevated on all plots before spring field work in 1996 with the application of triple super phosphate as 0-20-0 (elemental N-P-K), equivalent to 89 kg ha$^{-1}$ of elemental P.

Available N (AN) for the corn crop was defined as mineral sources of N available through additions by N fertilization and soil nitrate-N. AN does not include N that may be potentially released through mineralization of organic N during the growing season. Apparent N value (ANV) attributed to previous crop or N was calculated as

$$ANV = (\text{Actual Yield} - \text{Expected Yield}) \times 0.022 \qquad (2)$$

where Expected Yield = 45.45 × AN and Actual Yield was the measured crop yield. Positive deviations in ANV reflect corn yield in excess of the expected 45.45 kg of corn per kilogram of AN.

### Crop Measurements

Grain yields were measured with a Massey Ferguson MF 8-XP research plot combine (Kincaid Equipment Manufacturing*, Haven, KS) equipped with an electronic weigh bucket. On each plot, eight rows, each 30 m long (1/5 of the plot area), were harvested for grain yield. Subsamples of combine-harvested grain were retained for grain moisture, test weight, and N content. Grain moisture and test weight were measured with a Dickey-John GAC 2000 Grain Analysis Computer (Johnston, IA). Corn grain yields were adjusted to 15.5% moisture; soybean grain yields, to 10% moisture. Concentration of N and C in grain was measured using a Carlo Erba C, N, and S analyzer (Milan, Italy).

Samples to determine corn phytomass and N uptake were taken when the crop was mature and just prior to combine harvest. All plant material was cut from four rows, each 1 m long. Bundles were dried and weighed. Grain was separated from stover and weighed. Stover yield was determined as the difference between mass of bundle and mass of grain. Stover was shredded, subsampled, and ground for C and N analysis using a Carlo Erba C, N, and S analyzer. Soybean phytomass was sampled just before leaf drop.

### Soil Measurements

Samples for soil nitrate-N were collected in the fall or spring, depending on weather conditions. Samples for 1991–1996 crops were taken from 0- to 15-cm and 15- to 60-cm depths. After 1996, samples were taken to a depth of 120 cm at increments of 0 to 15 cm, 15 to 30 cm, 30 to 60 cm, 60 to 90 cm, and 90 to 120 cm. Three soil samples were taken randomly from each depth on each plot. Core diameter was 3.2 cm. Samples were dried at 40 °C immediately after sampling, ground through a 2 mm sieve, and subsampled. Measurements of nitrate-N in samples collected from 1991–1995 were made using a nitrate electrode procedure[11]. After 1995, nitrate-N was measured using 2 M KCl extraction and copperized Cd reduction column procedure[12].

In 1989, prior to the rotation study's establishment, 315 spatial samples of the experimental area were taken on a 30-by-30-m grid[13]. Samples were taken in August after a wheat crop. Intact soil cores 5.0 cm in diameter were taken from the Ap horizon and archived. Plow depth was about 20 cm. Kriging interpolation methods (Surfer v. 6.04, Golden Software, Golden, CO) of soil C and N obtained from the 1989 grid sampling were used to estimate initial C and N concentration at appropriate spatial coordinates of each 30-by-30-m plot within the grid sampled area. In June 2000, 6 cores were composited from 0- to 7.6-cm and 7.6- to 15-cm depths of each plot. Core diameter was 3.2 cm. Samples were taken from row positions.

Soil C and N were measured on soil samples collected in 1989 and 2000 using a LECO 2000 C-N analyzer (St. Joseph, MI). Both the LECO and the Carlo Erba analyzers use the dry combustion method for determination of total C and N in plant and soil samples. Soil samples from both 1989 and 2000 were dried at 60 °C, pulverized, and sieved through a 2-mm sieve. A subsample (approximately 30 g) of sieved soil was further ground on a roller mill and passed through a 0.5-mm sieve. A subsample (approximately 2 g) of soil that passed through a 0.5-mm sieve was dried at 100 °C for 2 hours and cooled in a desiccator, and approximately 0.4 g were transferred to nickel-lined ceramic combustion boats for total C-N analysis. Total C was considered to be total organic C because average (81 plots) soil pH of the top 15 cm was 6.5.

Statistical comparisons of all measurements were made using analysis of variance and multiple factor analysis of variance (MINITAB, Release 12, State College, PA). The split-plot arrangement within randomized blocks was such that factor 1 was tillage (whole plot).

## RESULTS AND DISCUSSION

### Crop Yield and N Use

Field experiments covered some of the wettest and coldest periods in the South Dakota climate record. Precipitation totals from 1991–1995 were the greatest in more than 100 years, and the 1992 and 1993 summers were the coldest consecutive summer seasons on record beginning in 1890 (Alan Bender, South Dakota State climatologist, Brookings, SD). In the first year of the experiment there was no significant difference in corn yield between rotations, but there was a significant response to N fertilizer. The highN treatment resulted in 2.2 Mg $ha^{-1}$ more grain than noN treatment. Results were important because they showed that our test site was responsive to N fertilizer (data not shown).

There was a significant ($p \leq 0.10$) corn-yield response to rotation. Average corn yield (11 years and 3 N treatments) under CSWA (Table 1) was about 35% greater than corn yield under CC and about 6% greater than corn yield under CS. Average (years and N treatments) fertilizer N applied under CSWA was significantly less (about 14%) than that applied to CC and CS. Autumn soil nitrate (average of years and N treatments) under CSWA was significantly greater than CC (41%) and CS (38%). There was a significant corn-yield response to N (Table 1). Average corn yield (average of rotations) under highN was 54% greater than corn yield under noN and 14% greater than corn yield under midN. There was a significant rotation × N interaction (Table 1) with respect to corn grain yield and available inorganic N.

We estimated usefulness of rotation and N to crop yield and expressed this calculation as ANV (Table 1). A negative number implies negative value, and a positive number implies added value. Positive deviations suggest that significant amounts of N were transformed (mineralized) from organic to inorganic forms during the growing season. Negative deviations show under utilization of N with respect to regional N fertilizer recommendation.

* Mention of trade names is for the benefit of the reader and does not constitute endorsement by the U.S. Department of Agriculture over other products not mentioned.

**TABLE 1**
**Average Corn Grain Yield, Applied Fertilizer N, Autumn Soil Nitrate-N, Available Inorganic N, and Apparent N Value (Based on Grain Yield) for 1990–2000***

| Rotation or N Treatment | Corn Grain Yield | Fertilizer N | Soil Nitrate-N | Available Inorganic N | Apparent N Value |
|---|---|---|---|---|---|
| | kg ha$^{-1}$ | | | | |
| Rotation (R) | | | | | |
| CC | 5111 | 66.7 | 48.9 | 115.6 | –5.6 |
| CS | 6499 | 66.0 | 50.0 | 116.0 | 23.7 |
| CSWA | 6908 | 57.3 | 69.0 | 126.4 | 22.2 |
| Fertilizer (N) | | | | | |
| HighN | 7343 | 123.9 | 60.8 | 184.7 | –26.6 |
| MidN | 6417 | 66.2 | 52.7 | 118.9 | 19.1 |
| NoN | 4758 | 0 | 54.4 | 54.4 | 47.8 |
| *p*-value year | 0.001 | 0.001 | 0.001 | 0.001 | 0.001 |
| *p*-value R | 0.001 | 0.022 | 0.011 | 0.044 | 0.001 |
| *p*-value N | 0.001 | 0.001 | 0.023 | 0.001 | 0.001 |
| *p*-value R × N | 0.002 | ns | ns | 0.047 | ns |

* Crop rotations were continuous corn (CC), corn–soybean (CS), and corn–soybean–wheat/alfalfa–alfalfa (CSWA). Corn was fertilized for a grain yield of 8.5 Mg ha$^{-1}$ (highN), 5.3 Mg ha$^{-1}$ (midN), and no N fertilizer (noN).

Indirectly, negative ANVs also suggest that N did not limit corn grain yield for our environmental conditions. Multifactor analysis of variance revealed significant differences among rotations and N fertilizer treatments (Table 1). Coefficient of variation (CV) was lowest (12%) under noN treatment and greatest (85%) under CC rotation.

Calculated ANV was based on regional N recommendations for corn in eastern South Dakota and as such provided an indicator of N-use efficiency. For simplicity, we associate enhanced yield with AN, but recognize that there are other factors responsible for improved yields beyond AN. We use the term *apparent nitrogen value* to acknowledge unknown enhancement factors. Average ANV for each of the rotations within N treatments show that ANV increased as N application decreased (Fig. 1). There was a high degree of variation in mean ANV. Consequently, one-way analysis of variance within fertilizer treatment revealed no significant ($p \leq 0.10$) differences in ANV among rotations. Lowest CVs (121%) and greatest benefits of rotation (measured as positive ANV, Fig. 1) were realized on noN plots under CS and CSWA rotation. On noN, cumulative ANV (1990–2000) was 284 kg ha$^{-1}$ under CC, 611 kg ha$^{-1}$ under CS, and 682 kg ha$^{-1}$ under CSWA. On highN, cumulative ANV (1990–2000) was –431 kg ha$^{-1}$ under CC, –183 kg ha$^{-1}$ under CS, and –266 kg ha$^{-1}$ under CSWA. Thus, a simplistic conclusion might be that the overall risk associated with inefficient N use might be minimized by using longer rotations (that include legumes) and reducing fertilizer N application.

We are uncertain if the N treatments used in this study provided for optimum or maximum corn grain yield within each rotation. Overall, there has been little difference among rotations in the amount of N fertilizer supplied in a given year. During the study, total AN (1990–2000) for the highN treatment was 2064 kg of N per hectare under CC, 2009 kg of N per hectare under CS, and 2024 kg of N per hectare under CSWA. With midN management, AN was 1287 kg of N per hectare under CC, 1292 kg of N per hectare under CS, and 1344 kg of N per hectare under CSWA.

Efficient use of N can minimize potential for groundwater contamination by leached nitrate. We found that increased N fertilization significantly increased $NO_3$-N to a depth of 3 m following 8 years of rotations and fertilizer treatments (samples were collected in 1998 and a complete data set is not shown). Under highN treatment, total $NO_3$-N in the top 3 m was 200 kg ha$^{-1}$ under CC, 143 kg ha$^{-1}$ under CS, and 134 kg ha$^{-1}$ under CSWA. Under noN treatment, total $NO_3$-N in the top 3 m was 73 kg ha$^{-1}$ under CC, 102 kg ha$^{-1}$ under CS, and 130 kg ha$^{-1}$ under CSWA. Measurement of soil $NO_3$-N in the top 3 m of soil provided a point-in-time evaluation of the quantity of $NO_3$-N remaining in the soil profile. We are uncertain if N has leached past the 3-m depth. Calculated ANV suggest that there was potential to lose N, especially under highN.

### Soil C and N

Research in the northern Corn Belt suggests that the balance of C in soil depends on crop yield. Larson et al.[14] showed that under conventional tillage, corn provided a net addition to soil organic C when the amount of cornstalk dry matter returned to the soil exceeded about 6.0 Mg ha$^{-1}$. Depending upon the ratio of corn stover to corn grain, this equates to a grain yield of about 6.3 Mg ha$^{-1}$. Inspection of Table 1 shows that average corn grain yield under all rotations was approximately at the C balance point

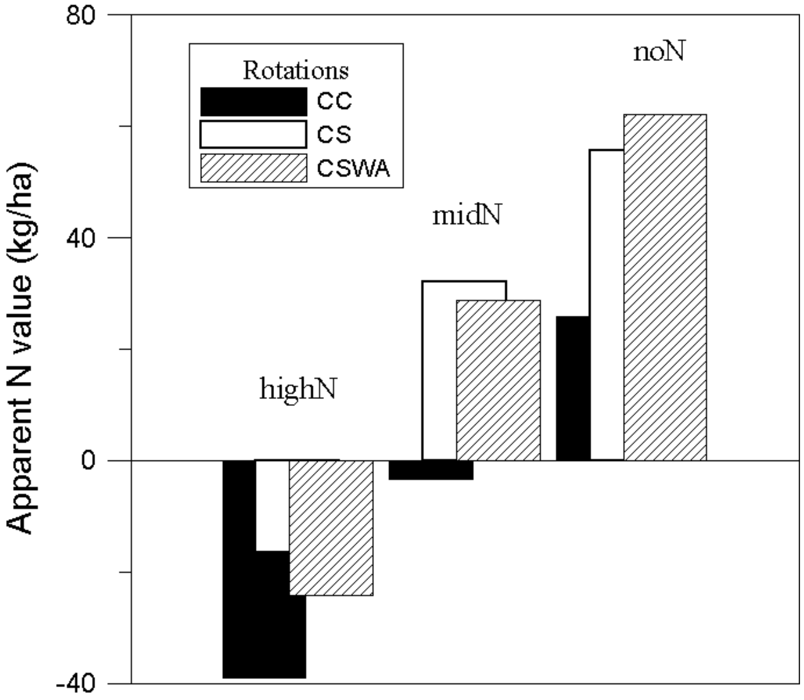

**FIGURE 1.** Average (11 years) apparent N value under rotations of continuous corn (CC), corn–soybean (CS), and corn–soybean–wheat/alfalfa–alfalfa (CSWA). N treatments (N applied according to regional fertilizer recommendations) were corn fertilized to achieve a yield goal of 8.5 Mg $ha^{-1}$ (highN), 5.3 Mg $ha^{-1}$ (midN), and no fertilizer N (noN). There were no significant differences in ANV among rotations within fertilizer treatments ($p \leq 0.10$, one-way analysis of variance).

suggested by Larson et al. In the past 10 years, total corn grain yield from CC plots under highN was 70.4 Mg $ha^{-1}$. This grain production produced 29.8 Mg of C per hectare that was returned to the soil. Our estimates are for aboveground plant material only. On plots under CC and highN, we measured a loss of 1.4 g $kg^{-1}$ (0.14%) organic C from the plow layer during the period 1989–2000 (results for each N treatment for each rotation are not shown in Table 1 or Table 2). On plots under CSWA and noN, 12.8 Mg

**TABLE 2**
**Total Soil C, Change in Soil C (2000–1989), Ratio of Total C to total N, and Change in C to N Ratio (2000–1989) in the Top 15 cm***

| Rotation or N Treatment | C Year 2000 | C Change 2000–1989 | C to N Ratio Year 2000 | C to N Change 2000–1989 |
|---|---|---|---|---|
| | g $kg^{-1}$ | | | |
| Rotation (R) | | | | |
| CSWA | 17.4 | –1.8 | 10.03 | –0.75 |
| CSr | 18.3 | — | 10.32 | 0.39 |
| CS | 17.6 | –1.7 | 10.22 | 0.02 |
| CC | 17.4 | –1.7 | 10.31 | 0.03 |
| Grass | 19.9 | 0.7 | 10.87 | 0.72 |
| Fertilizer (N) | | | | |
| HighN | 17.8 | –1.2 | 10.28 | 0.02 |
| MidN | 18.3 | –1.0 | 10.36 | 0.06 |
| NoN | 18.3 | –0.5 | 10.41 | 0.16 |
| *p*-value R | ns | 0.033 | 0.001 | 0.079 |
| *p*-value N | ns | ns | ns | ns |
| *p*-value R × N | ns | ns | ns | ns |

* Crops were corn–soybean–wheat/alfalfa–alfalfa (CSWA), corn–soybean with ridge tillage (CSr), corn–soybean with conventional tillage (CS), continuous corn (CC), and grass. Corn was fertilized for a grain yield of 8.5 Mg $ha^{-1}$ (highN), 5.3 Mg $ha^{-1}$ (midN), and no N fertilizer (noN).

of C per hectare were returned to the soil. On these plots we measured a loss of 1.7 g of C per kilogram (0.17%) from the plow layer during the 1989–2000 period. About 35% less tillage was used under CSWA than was used with CC.

We did not find a positive effect of crop rotation on soil C sequestration in any of the plots under conventional tillage. Rotations of CSWA, CS, and CC each lost about 1.7 g of C per kilogram from the plow layer (average of all N treatments within each rotation) during the period 1989–2000 (Table 2). In contrast, CSr rotation neither gained nor lost C, and plots under grass cover since 1989 gained about 0.7 g of C per kilogram in the top 15 cm.

Soil improvement is a slow process, and crop yield is a valuable indicator of soil condition, because the plant integrates across many biological, chemical, and physical soil properties. Within the framework of controlled laboratory soil-incubation studies designed to mimic field conditions during the growing season, we found that soil under CSWA had potential to mineralize about 56 kg $ha^{-1}$ more N than soil under CC[15]. We also found evidence that net N mineralization decreased about 8% with increased application of inorganic N. Soil organic matter is an important source of inorganic nutrients for plant production. Mineralizable N, a component of organic matter, is an important N source to crops. Corn grain yield has been greater under CSWA rotation compared with the yield under CC and CS rotations (Table 1).

Alfalfa in the CSWA rotation has had a positive and beneficial effect on soil condition by narrowing the soil C to N ratio during the 1989–2000 period. Table 2 shows a negative change in C to N ratio under CSWA, and this negative value represents a narrowing of the C to N ratio. Soil C to N has remained the same under CC and CS, and increased under CSr and grass (Table 2). Average C to N ratio of (typical values from 1997 crop) crop residues was about 14:1 for alfalfa, 89:1 for corn, and 46:1 for soybean. Benefit of legumes to soil N supply is well established[16], and these results show that a positive benefit on soil C to N was measurable following two complete (CSWA) rotations.

### CONCLUSION

Increased rotation length under CSWA increased corn yield while using about 14% less fertilizer N than that applied to CC and CS. Greatest ANV was realized under CSWA rotation with no additional inorganic N fertilizer. Increased N fertilization significantly increased $NO_3$-N to a depth of 3 m following 8 years of fertilizer treatments. Results show the necessity of tailoring N application rates to specific production levels when employing crop rotations to avoid excessive N leaching.

There are unresolved issues concerning the quantity of C that can actually be sequestered in soil and the time frame involved in that sequestration. On a yearly basis, CC captured significantly more C than CS or CSWA rotations did. CC returned 2.3 times as much C to the soil as a diversified 4-year rotation, but all rotations and N treatments under conventional tillage lost soil C during the 1989–2000 period. In contrast, plots under permanent grass cover since 1989 gained about 0.7 g $kg^{-1}$ C in the top 15 cm.

Our findings are applicable to other production areas in South Dakota as a guide to estimating reasonable rates of soil organic C accumulation or loss. With respect to corn production, our trials represent average corn production in South Dakota. Average corn grain yield for 1995–1999 was 6.8 Mg $ha^{-1}$ (107.8 bu per acre) for Brookings County and 6.5 Mg $ha^{-1}$ (103.2 bu per acre) for South Dakota[17]. Yield from our plots for 1995–1999 was 6.8 Mg $ha^{-1}$ (108.8 bu per acre). Therefore, the quantity of corn residue returned to the soil of our research plots under CC was representative of an average condition for South Dakota. Sustainability questions aside, CC may appear as a viable way to sequester C because of the large amount of organic material returned to the soil under corn. However, as evidenced by our soil C measurements from 1989–2000, all of the plant C returned annually under conventional tillage was subsequently lost to the atmosphere.

### ACKNOWLEDGEMENTS

We thank Max Pravecek, Biological Science Technician, and David Harris, Agricultural Science Research Technician Soils, for careful maintenance of the experimental plots. David Harris is also recognized for work in sample collection and technical laboratory analysis.

| | | |
|---|---|---|
| Received: | July | 11, 2001 |
| Revised: | August | 24, 2001 |
| Accepted: | August | 24, 2001 |
| Published: | September | 28, 2001 |
